# Electrophysiological Evidence of Local Sleep During Yoga Nidra Practice

**DOI:** 10.3389/fneur.2022.910794

**Published:** 2022-07-12

**Authors:** Karuna Datta, Hruda Nanda Mallick, Manjari Tripathi, Navdeep Ahuja, K. K. Deepak

**Affiliations:** ^1^Department of Physiology, All India Institute of Medical Sciences, New Delhi, India; ^2^Department of Sports Medicine, Armed Forces Medical College, Pune, India; ^3^Faculty of Medicine and Health Sciences, SGT University, Gurugram, India; ^4^Department of Neurology, All India Institute of Medical Sciences, New Delhi, India

**Keywords:** delta power, electrophysiological characterization, local sleep, sleep quality, *yoga nidra*

## Abstract

**Background and Objectives:**

*Yoga nidra* is a technique sages use to self-induce sleep. Classically, sleep is characterized by three cardinal electrophysiological features, namely, electroencephalography (EEG), electromyography (EMG), and electrooculography (EOG). As the literature on electrophysiological characterization of *Yoga nidra* is lacking, it is not known whether it is a sleep or awake state. The objective of the study was to electrophysiologically characterize yoga nidra practice.

**Materials and Methods:**

Thirty subjects underwent five initial supervised yoga nidra sessions and then continued practice on their own. The subjects completed their sleep diaries for 2 weeks before and during the intervention. The electrophysiological characterization was done after 2 weeks of yoga nidra practice using 19 EEG channels polysomnography for pre-*yoga nidra, yoga nidra* practice and post-*yoga nidra*. Polysomnographic data were scored for sleep-wake stages as per standard criteria. Power spectral density (PSD) was calculated from various frequency bands in different time bins. EEG data were grouped by areas, namely, central, frontal, prefrontal, parietal, temporal, and occipital in time bins. Sleep diary parameters were also compared for pre-post-yoga nidra training.

**Results:**

After 2 weeks of yoga nidra practice, awake was scored throughout the session (*n* = 26). PSD results (mean difference in dB between different time bins; *P* value) showed significant changes. When compared to pre-yoga nidra, there was an increase in delta power in the central area (1.953; *P* = 0.033) and a decrease in the prefrontal area (2.713; *P* = 0.041) during yoga nidra. Sleep diary showed improvement in sleep duration (*P* = 0.0001), efficiency (*P* = 0.0005), quality (*P* = 0.0005), and total wake duration (*P* = 0.00005) after 2 weeks of practice.

**Interpretations and Conclusions:**

*Yoga nidra* practice in novices is electrophysiologically an awake state with signs of slow waves locally, often referred to as local sleep.

**Clinical Trial:**

Clinical Trial Registry of India, http://www.ctri.nic.in/Clinicaltrials/pmaindet2.php? trialid = 6253, 2013/05/003682.

## Introduction

Ancient literature states that *yogis* or sages could go through a special kind of sleep called yogic sleep or *yoga nidra* (1). The method of learning *yoga nidra* was not documented and was taught by the teacher only to his disciple until *Swami* Satyananda Saraswati, a renowned teacher from Bihar School of Yoga, Munger, Bihar, India, laid down the concept of *yoga nidra* and the technique of doing it, following simple instructions using his book “*Yoga Nidra*” (2).

*Yoga nidra* has been studied in long-term meditators using functional magnetic resonance imaging (3) and positron emission tomography (PET) (4). *Yoga nidra* state of a meditator was first recorded in 1977, by Dr Elmer Green (2) using EEG. The meditator had made a predetermined sequence set of 5 min for the various stages of sleep-wake and was able to demonstrate them, electrophysiologically while performing *yoga nidra*. This implies that the state of long-term meditators who are also yoga nidra practitioners, unlike novices can be predetermined by established long-term meditators themselves. A recent review by Thomas and Cohen described years of practice as a problem in meditation research (5), since research findings keep changing as the meditator puts in more years of practice. A concept of “therapeutic yoga” is emerging as a treatment modality for patients. To understand the effects of a particular yoga practice it is better to conduct studies on novices rather than meditators. It is also required to lay out the module clearly in a standardized format.

*Yoga nidra* has been tried in patients with menstrual abnormalities, diabetes, anxiety, and depression, but it effects on sleep are not well known. Also, what happens during this practice is not documented. “Datta et al.” reported the use of *yoga nidra* in insomnia using a therapeutic model of *yoga nidra* which was developed after inputs from yoga instructors, sleep medicine experts, and sleep scientists (6). Since *yoga nidra* is an acceptable therapeutic option, there was a felt need to characterize it electrophysiologically in subject volunteers to understand the practice and its effect. The objective of this study was to electrophysiologically characterize yoga nidra practice in novices.

## Materials and Methods

Thirty healthy adult volunteers were recruited for the electrophysiological characterization of *yoga nidra*. The study was approved by the Institutional Ethical Committee of the All India Institute of Medical Sciences, New Delhi, India, with reference number IESC/T-394/02.11.2012. The study was a part of the registered CTRI trial (CTRI/2013/05/003682) conducted from 2012 to 2017 at a premier institute in India. The present study describes one of the objectives of the trial. The recruitment process was started by local advertisement. The study was of a pre-post intervention design.

Because of its description as yogic sleep, the characterization was done using digital polysomnography. A baseline 2 weeks sleep diary was used to screen subjects.

### Subjects

After screening, chosen subjects were explained the nature of the study and informed consent was obtained.

### Inclusion and Exclusion Criteria

Healthy adult volunteers, following their usual sleep-wake schedule during the study were included. To rule out short and long sleepers, a baseline sleep diary of 2 weeks was used to exclude volunteers sleeping less than 300 min and more than 540 min (7).

Subjects with a morning circadian preference (those who are relatively more alert and attentive in the morning) were included based on their morningness–eveningness scores of more than 59/86 (8). Care was taken to exclude subjects with an evening circadian preference.

*Yoga nidra* is done with an alert mind such that one can follow the instructions in the audio tape for practice. The sessions were thus planned for the morning time.

Subjects with a history of any acute illnesses such as viral fever, cold, upper respiratory tract infection, or gastroenteritis in the preceding one month that were likely to cause sleep disturbances were excluded. Subjects with a history of sedatives, systemic steroids, and diagnosis of pregnancy were also excluded from the study.

### *Yoga nidra* Session

The method of doing *yoga nidra* involves seven steps, namely, preparation, *samkalpa* (*sankalpa* = idea or notion formed in the heart or mind), body part awareness or rotation of consciousness, breath awareness, feeling and sensation, visualization, and ending of practice (2).

The sessions were done using the therapeutic model (6). *Yoga nidra* session was done during the morning at a time when the subject was most alert. The same time was used for supervised sessions for the next 5 days. During the conduct of each supervised session, the investigator followed the “guidelines for observer” developed for the practice (9). Only one subject was studied on one day. After the supervised sessions, the subject was asked to do the session every day in the morning on their own using the same audio used during practice. They came back after starting *yoga nidra for* regular *follow*-*up*.

Electrophysiological characterization of yoga nidra practice was done by recording the session at the end of 2 weeks of *yoga nidra* practice.

LDxN ©Philips Respironics (USA) digital PSG system was used for electrophysiological recording during the *yoga nidra*. To characterize *yoga nidra*, standard PSG was used as per recommended guidelines of the American Academy of Sleep Medicine (AASM) (10) during the recording session.

The subjects were instructed to report in the morning after a head bath with no oil. After standard bio-calibration, baseline or pre-*yoga nidra* parameters were recorded by alternately closing and opening the eyes for one min each for a total of four times, i.e., a total of eight min. Following this, the subject was asked to follow the *Yoga nidra*© CD of Bihar School of Yoga, Munger, Bihar, India. After this, the subject was again asked to open and close their eyes alternately for 1 min each for a total of 8 min i.e., four times eyes open and eyes closed. The exact time mark of bio calibration, eyes closed, eyes open, and the start of *yoga nidra* was marked simultaneously on the online recording. The time for each recording protocol was thus eight min baseline consisting of pre *yoga nidra* of alternate eyes closed and eyes open for 1 min each, four times, 27.2 min of *yoga nidra* following *yoga nidra*© CD and eight min post-*yoga nidra* eyes open and closed, i.e., a total of approximately 44 min excluding bio calibration. At the end of the recording, the subject was asked to enumerate the different phases of *yoga nidra*. Subjects who were not able to recall the various parts of *yoga nidra* after the session were removed from the analysis since it meant non-compliance with the intervention.

### PSG Data Analysis

PSG files were coded with a unique number. PSG data including EEG, EOG, and EMG were analyzed epoch wise for sleep-wake staging using AASM guidelines (10). After AASM scoring of sleep-wake, 19 channel EEG data (sampling rate 500 Hz, LFF 0.3 Hz, and HFF 196 Hz) were further analyzed. Maximum electrode impedance acceptable was kept at 5 kΩ.

The pre-processing of EEG data was done on G3 Sleepware©, Philips Respironics, USA, where each acquisition was scanned manually epoch wise for artifacts and was carefully marked on raw data. The event list was prepared from the start of the baseline, i.e., pre-*yoga nidra* eyes closed. Since the eyes were open, eyes closed of both pre- and post-*yoga nidra*, and the start of *yoga nidra* were marked on each recording while the online recording was being done, the time bins for analysis were used from these time points. The time bins for EEG data analysis are shown in Supplementary Table S1.

Nineteen-channel EEG data was extracted from the EDF (European Data Format) file of G3 Sleepware©, Philips Respironics, USA, using AWA© software, Tokyo, Japan.

The EEG data were imported in EEG Lab (13_3_2b)© (11) using MATLAB© 7.12.0 (R2011a) (MathWorks. Inc., USA). The artifacts that were marked initially were now manually removed after noting the time from the event list.

The low cut filter was set at 1 Hz, high cut filter at 90 Hz and the notch filter at 50 Hz. Independent component analysis (11) was done for all acquisition files using “*Runica extended”*. Power Spectral Density (PSD) was calculated using the “*spectopo*” function of EEG Lab with a 512 ms window (12). The inputs for this function were the EEG data with time bins as required for analysis and frequency sampling rate. The PSD values were calculated for all 19 channels. The outputs of this function were power spectra in dB and frequencies in Hz. A formula was used to group PSD values in frequency bins and finally, the data was calculated in the following frequency bands—Delta (1–4 Hz); Theta 1 (4–6 Hz); Theta 2 (6–8 Hz); Alpha 1 (8–10 Hz); Alpha 2 (10–12.5 Hz) and Beta (13–30 Hz). The PSD data of 19 electrode positions were then rearranged region wise into central, frontal, pre-frontal, parietal, temporal, and occipital, i.e., an average of C3, C4, and Cz for central region; F3, F4, F7, F8, and Fz for frontal region; Fp1 and Fp2 for pre-frontal region; P3, P4, and Pz for parietal region; T3, T4, T5, T6 for temporal region; and O1 and O2 for occipital region for each of the 21 EEG time-bins. Data in these six regions was compiled with the EEG Time-bins from 1 to 21 for all the six frequency bands. The artifact was manually marked on PSG data and was removed using the start and end time of the artifact in the EEGlab.

### Sleep Diary

Subjects were encouraged to fill in the sleep diary till 4 weeks after intervention. Subjective Time in Bed (TIB), Total Sleep Time (TST), Total Wake Duration (TWD = TIB-TST), sleep efficiency = (TST/TIB) ^*^100 and sleep quality were calculated (6). Baseline 2 week data of sleep diary parameters were compared with the last 2 weeks of a sleep diary, i.e., starting from 2 weeks after yoga nidra practice intervention.

## Results

Thirty-five subjects were screened for eligibility and 30 subjects in the age group of 18–60 years (mean ± SD = 35.53 ± 8.69 years) were recruited and completed the study, of which 15 were males and 15 were females. They were all apparently healthy volunteers and right-handed individuals.

### Electrophysiological Recording Data Using PSG During *Yoga nidra*

Thirty subjects were recruited for the study. Two weeks after the five supervised sessions, the subjects were asked to report for electrophysiological recording. During these 2 weeks, all the subjects practiced yoga nidra every day. Only 27 subjects reported for recording after 2 weeks of training and three did not due to personal reasons such as job constraints, bad weather, and illness. One subject was excluded from the analysis as he was non-compliant; thus, the remaining 26 files were analyzed. PSG data were analyzed using AASM criteria (10). Using this criterion, all the recordings showed the entire *yoga nidra* practice as awake. A report of the subject obtained after scoring using AASM guidelines is shown in Supplementary Figure 1, which shows the analysis as awake. EEG data was taken from the PSG files for further analysis.

**Figure 1:**
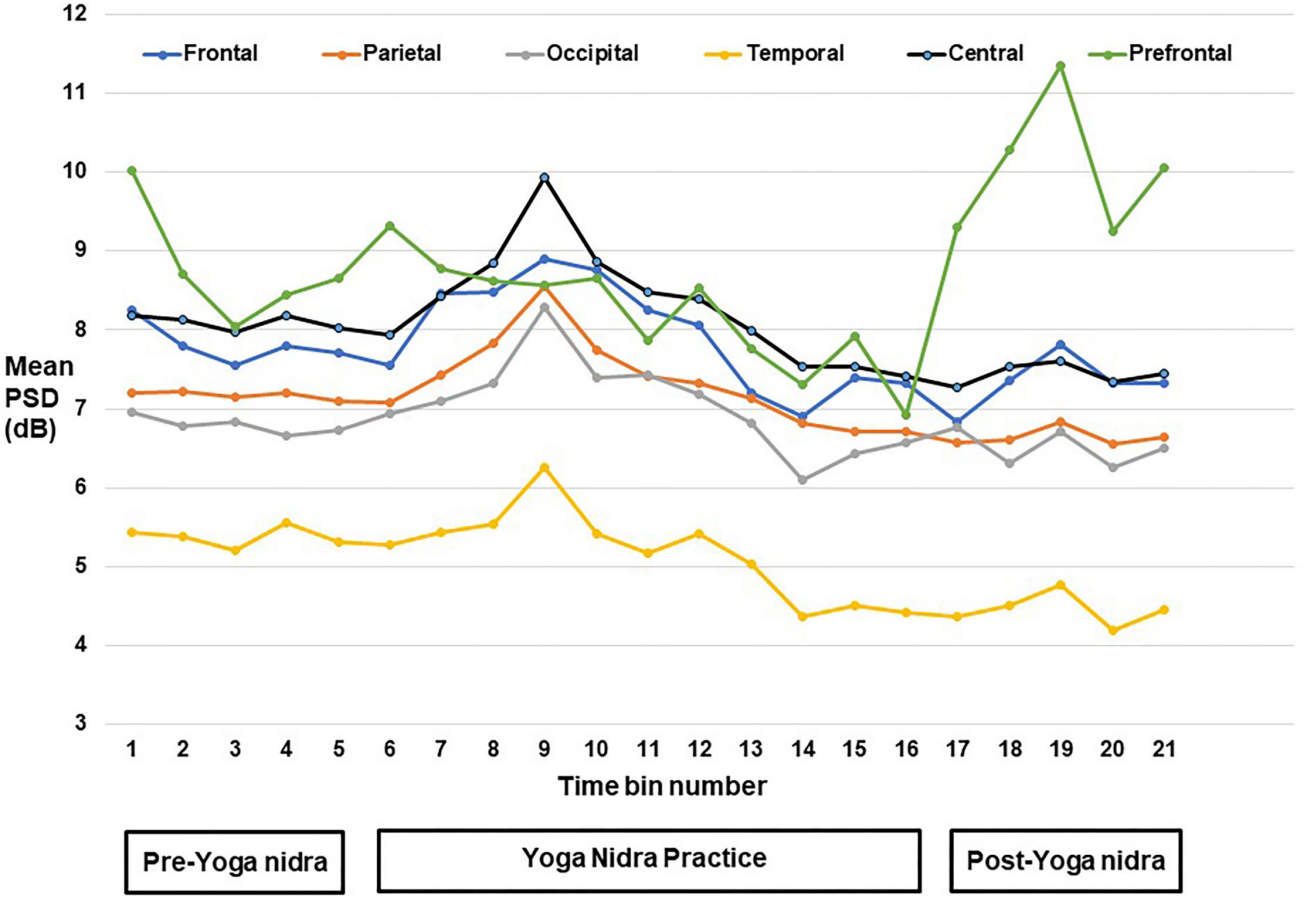
Mean power spectral density (PSD) values of delta frequency band at pre-frontal, frontal, central, parietal, temporal, and occipital regions.

**Figure 2:**
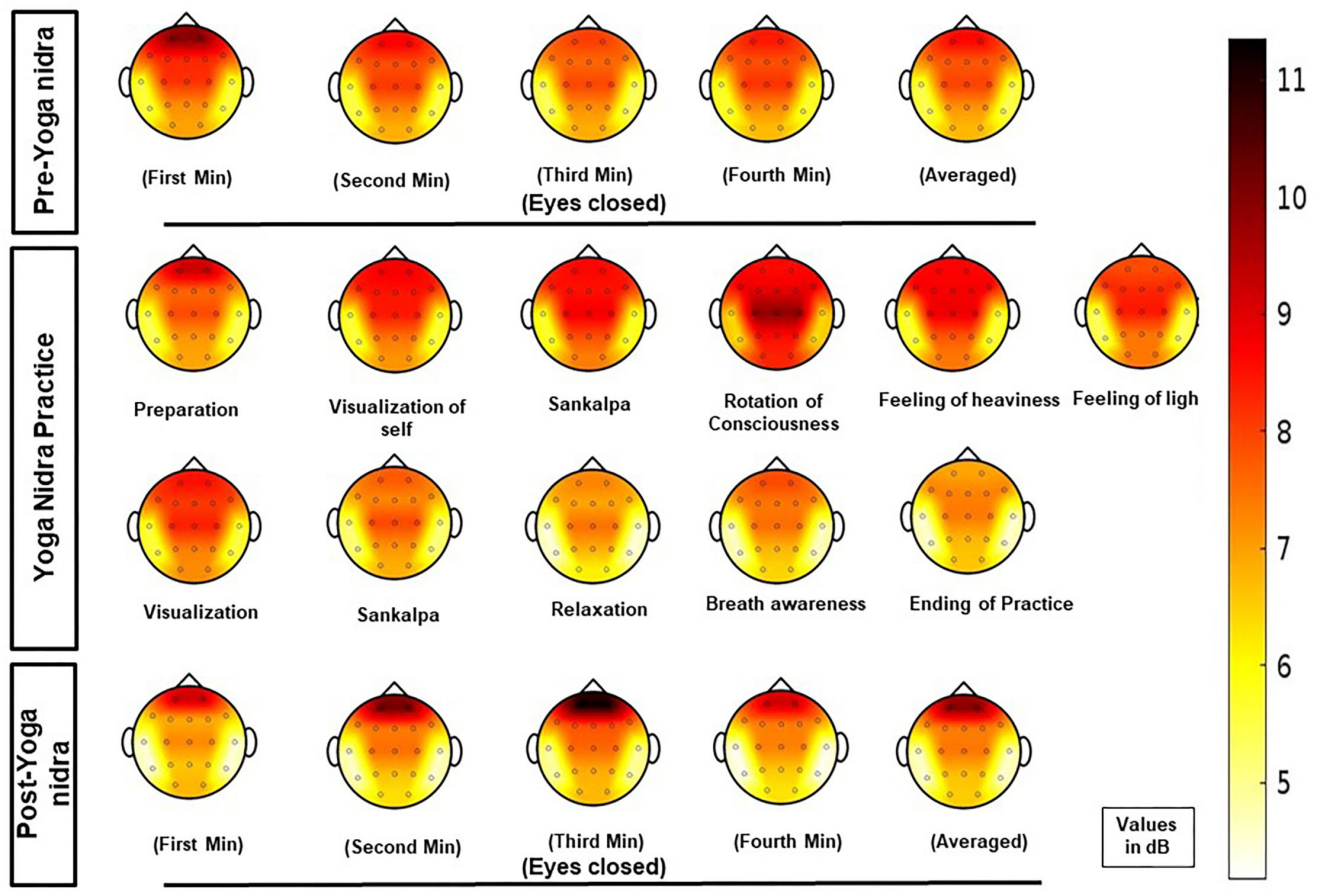
Topo plots of mean power spectral density (PSD) values of delta frequency band at all regions (pre-frontal, frontal, central, parietal, temporal, and occipital) during the *yoga nidra* recording session, i.e., pre-yoga nidra (just before the practice), during yoga nidra practice and post-yoga nidra (just after the practice).

PSD calculation from EEG: For each of the six regions, namely, frontal, central, pre-frontal, parietal, occipital, and temporal, MANOVA analysis was done with Sidak *post hoc* tests, and EEG Time-bins from one to 21 showed many combinations for the frequency bands at the six regions; thus, results of significant *post hoc* tests for delta, theta-1, alpha-1, and alpha-2 frequency bands are tabulated in Supplementary Tables S2–S5 respectively. Though using AASM criteria, the epochs were scored as awake. EEG analysis of PSD results (mean difference in dB between different time bins; *P* value) showed significant changes. Increased delta power during *yoga nidra* at the central area (1.953; *P* = 0.033) and reduction in prefrontal area (2.713; *P* = 0.041) was seen when compared to pre yoga nidra. The detailed significant results of the delta frequency band analysis are shown in Supplementary Table S2. Figure 1 shows the mean PSD values of delta frequency for all six regions. Figure 2 shows the topo plots of mean PSD values of the delta frequency over all the regions during the yoga nidra recording session, namely, pre-yoga nidra (just before the practice), during yoga nidra practice and post-yoga nidra (just after the practice). Significant results of theta-1, alpha-1, and alpha-2 frequency bands are given in Supplementary Tables S3–S5, respectively. Theta-2 and Beta frequency bands did not show any significant results.

### Sleep Diary

Sixteen subjects submitted completed diaries after 4 weeks of intervention. Two weeks of baseline were compared with the third- and fourth-week of intervention. Table 1 shows the sleep diary parameters of these subjects. There was a significant improvement in subjective TST (*P* = 0.0001), sleep efficiency (*P* = 0.0005), TWD (*P* = 0.00005), and sleep quality (*P* = 0.01) after yoga nidra practice. There was no significant change in TIB.

**Table 1:** Sleep diary parameters of subjects.

**Sr No**.	**Sleep diary parameters (*n =* 16)**	**Mean**	**SD**	***p* value**
1	Time in bed- baseline (min)	451.67	55.46	0.25
2	Time in bed- post intervention (min)	453.97	48.61	
3	Total sleep time- baseline (min)	443.48	54.46	0.0001
4	Total sleep time- post intervention (min)	450.90	50.11	
5	Total wake duration*- baseline (min)	9.27	14.00	0.00005
6	Total wake duration*- post intervention (min)	3.88	7.84	
7	Sleep quality rating- baseline (out of 10)	7.45	1.09	0.01
8	Sleep quality rating- post intervention (out of 10)	8.26	0.65	
9	Sleep efficiency- baseline (percentage)	98.10	3.11	0.0005
10	Sleep efficiency- post intervention (percentage)	99.09	1.85	

^*^Total wake duration = Sleep onset latency + Wake after sleep onset. ‘p value' signifies the comparison between baseline and post intervention parameters.

## Discussion

In *yoga nidra*, supine posture is adopted and a system of *pratyahara* is used in which withdrawal of senses is done, which is similar to the physiological initiation of sleep where one assumes supine posture and sensory withdrawal, or sensory attenuation occurs. The subjects did not show any electrophysiological evidence of sleep during the practice as analyzed using AASM guidelines, and hence *yoga nidra* cannot be equated to natural sleep in subjects. However, *yoga nidra* caused significant changes in PSD values as compared to the pre-yoga nidra state, implying that the intervention does alter the EEG. Post-*yoga nidra* was also significantly different from *yoga nidra* and pre-yoga nidra state.

After baseline, *yoga nidra* practice during rotation of consciousness caused a significant increase in delta power without an increase in power of theta-1 and theta-2 in the central region. This was in contrast to sleepiness, where both delta and theta power increased (10). A reduction in alpha power was seen in occipital and parietal areas during the *yoga nidra* practice as compared to baseline. In Qigong practice (13), *Santhi kriya* (14), meditators (15), and in novices (16) after *kundalini* yoga (17), an increase in alpha power was seen implying *yoga nidra* to be different from meditation.

Local sleep has been demonstrated in humans after sleep deprivation (18), and after cognitive tasks (19). The study by Bernardi et al. showed an increase in theta following sleep deprivation in frontal and posterior brain areas (18). The evidence of local sleep with learning in the human brain was first reported by Huber et al. where local sleep after learning improved task performance (20). The best-established marker of local sleep is slow-wave EEG activity. In our study, there was a significant increase in delta power differentially at the central and parietal areas during yoga nidra practice, implying local dominance of EEG slow waves (21) during an electrophysiologically awake state, which can only be explained as local sleep. Prefrontal areas showed a reduction in delta power as compared to pre- and post-*yoga nidra*, implying that different areas behave differently during the practice of *yoga nidra*. Changes in the temporal area seen maybe because of auditory stimulus during *yoga nidra* practice (22).

Since the subject is electrophysiologically not asleep but awake during *yoga nidra*, yet EEG showed an increase in power of delta and reduction of alpha-1 in certain areas, is it an “aware sleep” since the prefrontal area is active? Is it a kind of state which gives benefits of sleep despite being physiologically awake as in animals with hemispheric sleep like dolphins? Such a kind of unique state has been described by experienced yoga nidra practitioners.

Local sleep is attributed to the past state of neurons, after increased active state, metabolite accumulation, or the production of sleep promoting substances, causing sleep generating neuronal mechanisms to get activated directly or indirectly (23). This does not explain its occurrence in our subjects as the subject was resting in pre-yoga nidra.

Post-*yoga nidra* practice showed a significant reduction in power in the delta band as compared to the practice of *yoga nidra* in all the regions, i.e., frontal, parietal, temporal, central, and occipital except the prefrontal where both theta-1 and delta significantly increased. This may be due to activity in this area during the yoga nidra session (23). The central region showed a significant increase in theta-1 power, implying slowing to be continuing although delta power was reduced. There was an increase in alpha-1 and alpha-2 power in central, parietal, and temporal areas. The occipital region showed a reduction in delta and theta-1, with an increase in alpha-1 and alpha-2 in post-*yoga nidra* as compared to *yoga nidra*. This may be because, in post-*yoga nidra*, the subject was asked to only close their eyes without any task during eye closure. While in *yoga nidra*, though the eyes were closed the subject was constantly attentive to the instructions and performing the task mentally. This can also probably explain the reduction in alpha power during *yoga nidra* practice which becomes significantly increased after *yoga nidra*-like alpha block phenomena during *yoga nidra* practice.

An increase in alpha power post-*yoga nidra* may be associated with reduced sensory input and alpha waves are extremely sensitive to stimulus presentation and expectation (24) and the disengagement of the parietal cortex from object selection causing increased alpha (25) is reported. A positive correlation is seen between thalamic activity and alpha waves (26). Lack of auditory stimulus may have reduced the sensory input in the post-*yoga nidra* phase.

In *yoga nidra* practice, there is a phase in which rotation of consciousness to various parts of the body is done, along with visual imagery of clouds, houses, flowers, etc. Considering that visualization is involved in the practice (27, 28), we did not find any slowing in the temporal area. On the contrary, the temporal area showed a reduction of power in delta and theta-1 during and after *yoga nidra* practice (29). This could also be due to auditory input processing and attention, as previously discussed.

Although increased delta power is seen over some areas during the *yoga nidra* session, does it reduce the sleep debt which usually occurs from sleep deprivation? It is still unknown how do local sleep phenomena or local slow waves behave on the sleep debt. The subjects showed a significant improvement in subjective quality of sleep, TST, and wake duration. High cardiac vagal control is related to better subjective sleep quality (30). Yoga practice in the morning has been found to increase parasympathetic drive at night (31) causing sleep to be more restorative. The probable mechanism may be reduced arousal along with parasympathetic dominance. Some studies have found an increase in dopamine release during *yoga nidra* in meditators and highlighted it as being associated with increased theta power. Dopamine release in the striatum was found to be associated with reduced readiness and played an important role in gating of sensory inputs as proposed by Horvitz in 2002 (32). *Yoga nidra* is a pratyahara technique in which the subject is made to withdraw from the senses and observe as a neutral observer, and hence differs from mere relaxation. Enhanced sensory experience with the withdrawal of senses has been linked to dopamine release in the ventral striatum (4) suggesting dopamine may be active in regulating consciousness. The increased dopaminergic tone in the striatum can affect the frontal subcortical circuits, affecting the prefrontal cortex and cingulate cortex. We were unable to do sleep studies for subjects and so the exact effect of a *yoga nidra* session done during the day on the night PSG could not be established in subjects.

Since the mode of training for *yoga nidra* is using an instructional tape for novices, the exclusion of short sleepers was done using a baseline sleep diary to avoid altered processing of auditory signals due to reduced sleep duration (7, 33).

In our study, we cannot comment on the time that these trait effects lasted, but we did see that post-*yoga nidra* EEG was distinctly different from baseline. It might be possible to understand these changes in subjects with a longer duration of practice.

The study used PSD rather than Fast Fourier Transform values and multiple sampling of the practice was done to find out the effects of the entire practice on the continuum. However, there are some limitations. Authors have documented short-term effects of the yoga nidra practice in novices keeping “Therapeutic Yoga” concept in mind (5), however, long-term duration of yoga nidra practice on a larger sample may give further information. Long-term yoga nidra practitioners also follow meditation, hence, subjects only with a long duration of yoga nidra practice may be planned to characterize long-term effects of yoga nidra in its practitioners.

In our study, we used prospective 2 weeks sleep diary data which though a recommended criteria for assessing sleep but is subjective in nature. An objective test of overnight PSG is the gold standard but could not be done, being costly and time-consuming and often normal healthy controls refrained from spending one night in the lab.

We have for the first time shown induction of local sleep by practice of Yoga nidra along with its electrophysiological characterization which will add a new dimension to mental relaxation practice. The local increase in slow waves following yoga nidra practice may be important to help learning as shown by Huber et al. (20). More studies using yoga nidra practice are required to establish its role in enhancing task-specific performance.

## Conclusions

*Yoga nidra* practice in apparently healthy subjects appears to be an awake state, but with local sleep changes in some areas of the brain. Such signs of the local slow wave during a waking state have been described as local sleep in animals and humans. The electrophysiological study reveals a unique feature of *yoga nidra* where some parts of the brain exhibit sleep while the rest are in an awake state. Its practice in the morning improves subjective sleep quality at night.

## Data Availability Statement

The original contributions presented in the study are included in the article/Supplementary Material, further inquiries can be directed to the corresponding author/s.

## Ethics Statement

The studies involving human participants were reviewed and approved by Institutional Ethical Committee with reference number IESC/T-394/02.11.2012. The study was a part of the registered CTRI trial (CTRI/2013/05/003682) conducted in India. The patients/participants provided their written informed consent to participate in this study.

## Author Contributions

All authors listed have made a substantial, direct, and intellectual contribution to the work and approved it for publication.

## Conflict of Interest

The authors declare that the research was conducted in the absence of any commercial or financial relationships that could be construed as a potential conflict of interest.

## Publisher's Note

All claims expressed in this article are solely those of the authors and do not necessarily represent those of their affiliated organizations, or those of the publisher, the editors and the reviewers. Any product that may be evaluated in this article, or claim that may be made by its manufacturer, is not guaranteed or endorsed by the publisher.
