# Supplementary material for: Electrophysiological Evidence of Local Sleep During Yoga Nidra Practice
Source: Front Neurol. 2022 Jul 12;13:910794. doi: 10.3389/fneur.2022.910794 (PMC9315270; doi:10.3389/fneur.2022.910794)
Supplement: Supplementary file 1 [file Table_1.docx]

**Table S1: Time bins for analysis of EEG from PSG data**

| **Components of recording session** | **Time-bin number and details of the time bin** | | **Time** |
| --- | --- | --- | --- |
| Pre-YN practice | 1 | BLEC1 | 1 minute |
|  | 2 | BLEC2 | 1 minute |
|  | 3 | BLEC3 | 1 minute |
|  | 4 | BLEC4 | 1 minute |
|  | 5 | Average of BLEC |  |
| YN* practice | 6* | YN1 - Preparation | 60–90 seconds |
|  | 7* | YN2 – Visualization of self | 190–330 seconds |
|  | 8* | YN3 - Sankalpa | 360–390 seconds |
|  | 9* | YN4 – Rotation of consciousness | 739–789 seconds |
|  | 10* | YN5 – Feeling of heaviness | 930–1020 seconds |
|  | 11* | YN6 – Feeling of lightness | 1035–1115 seconds |
|  | 12* | YN7 – Visualization | 1230–1315 seconds |
|  | 13* | YN8 – Relaxation | 1329–1380 seconds |
|  | 14* | YN9 – Sankalpa | 1455–1485 seconds |
|  | 15* | YN10 – Awareness of breath | 1490–1522 seconds |
|  | 16* | YN11 – Ending of practice | 1530–1560 seconds |
| Post-YN practice | 17 | PYEC1 | 1 minute |
|  | 18 | PYEC2 | 1 minute |
|  | 19 | PYEC3 | 1 minute |
|  | 20 | PYEC4 | 1 minute |
|  | 21 | PYEC average |  |
| *Parts of *Yoga nidra* from the *Yoga nidra***^©^** CD and time correlates to the start of *yoga nidra* from CD, YN: *Yoga nidra*, BLEC: Baseline eyes closed, PYEC: Post *Yoga nidra* eyes closed | | | |

**Table S2**- Significant post hoc results in Delta Frequency Band during different EEG time bins from 1-21 i.e. during the entire *yoga nidra* (YN) recording session including pre *yoga nidra* or Baseline (EEG time bins 1-5), *yoga nidra* (EEG time bins 6-16) and post *yoga nidra* (EEG time bins 17- 21) in the subjects at various regions.

| S. no | Two EEG Time-bins (from 1 -21) in which significant result was found | | Mean Difference (^#^PSD values in dB between EEG time bins) | P- value |
| --- | --- | --- | --- | --- |
| Frontal Delta | | | | |
| 1 | *9- YN4 – Rotation of consciousness | 17- Post YN Eyes Closed 1 | 2.073 | 0.043 |
| Central Delta | | | | |
| 2 | 2-Baseline Eyes Closed 2 | *9- YN4 – Rotation of consciousness | -1.806 | 0.046 |
| 3 | 3-Baseline Eyes Closed 3 | *9- YN4 – Rotation of consciousness | -1.953 | 0.033 |
| 4 | 5- Baseline Eyes Closed Average | *9- YN4 – Rotation of consciousness | -1.915 | 0.034 |
| 5 | *6- YN1 - Preparation | *9- YN4 – Rotation of consciousness | -2.007 | 0.028 |
| 6 | *13- YN8 – Relaxation | *9- YN4 – Rotation of consciousness | -1.946 | 0.036 |
| 7 | *14- YN9 – Sankalpa | *9- YN4 – Rotation of consciousness | -2.402 | 0.011 |
| 8 | *15- YN10 – Awareness of breath | *9- YN4 – Rotation of consciousness | -2.406 | 0.01 |
| 9 | *16- YN11 – Ending of practice | *9- YN4 – Rotation of consciousness | -2.518 | 0.007 |
| 10 | 17- Post YN Eyes Closed 1 | *9- YN4 – Rotation of consciousness | -2.661 | 0.003 |
| 11 | 18- Post YN Eyes Closed 2 | *9- YN4 – Rotation of consciousness | -2.404 | 0.008 |
| 12 | 19- Post YN Eyes Closed 3 | *9- YN4 – Rotation of consciousness | -2.325 | 0.01 |
| 13 | 20- Post YN Eyes Closed 4 | *9- YN4 – Rotation of consciousness | -2.588 | 0.004 |
| 14 | 21- Post YN Eyes Closed Average | *9- YN4 – Rotation of consciousness | -2.495 | 0.006 |
| Parietal Delta | | | | |
| 15 | *9- YN4 – Rotation of consciousness | *15- YN10 – Awareness of breath | 1.830 | 0.04 |
| 16 | *9- YN4 – Rotation of consciousness | *16- YN11 – Ending of practice | 1.830 | 0.04 |
| 17 | *9- YN4 – Rotation of consciousness | 17- Post YN Eyes Closed 1 | 1.971 | 0.023 |
| 18 | *9- YN4 – Rotation of consciousness | 18- Post YN Eyes Closed 2 | 1.939 | 0.026 |
| 19 | *9- YN4 – Rotation of consciousness | 19- Post YN Eyes Closed 3 | 1.703 | 0.05 |
| 20 | *9- YN4 – Rotation of consciousness | 20- Post YN Eyes Closed 4 | 1.999 | 0.021 |
| 21 | *9- YN4 – Rotation of consciousness | 21- Post YN Eyes Closed Average | 1.903 | 0.028 |
| Temporal Delta | | | | |
| 22 | *9- YN4 – Rotation of consciousness | 20- Post YN Eyes Closed 4 | 2.071 | 0.032 |
| Occipital Delta | | | | |
| 23 | *9- YN4 – Rotation of consciousness | *14- YN9 – Sankalpa | 2.184 | 0.025 |
| 24 | *9- YN4 – Rotation of consciousness | 18- Post YN Eyes Closed 2 | 1.975 | 0.037 |
| 25 | *9- YN4 – Rotation of consciousness | 20- Post YN Eyes Closed 4 | 2.030 | 0.032 |
| Prefrontal Delta | | | | |
| 26 | 2-Baseline Eyes Closed 2 | 19- Post YN Eyes Closed 3 | -2.641 | 0.043 |
| 27 | 3-Baseline Eyes Closed 3 | 19- Post YN Eyes Closed 3 | -3.317 | 0.013 |
| 28 | 4-Baseline Eyes Closed 4 | 19- Post YN Eyes Closed 3 | -2.905 | 0.026 |
| 29 | 5- Baseline Eyes Closed Average | 19- Post YN Eyes Closed 3 | -2.694 | 0.039 |
| 30 | *8- YN3 - Sankalpa | 19- Post YN Eyes Closed 3 | -2.739 | 0.039 |
| 31 | *9- YN4 – Rotation of consciousness | 19- Post YN Eyes Closed 3 | -2.793 | 0.036 |
| 32 | *10- YN5 – Feeling of heaviness | 19- Post YN Eyes Closed 3 | -2.693 | 0.043 |
| 33 | *11- YN6 – Feeling of lightedness | 19- Post YN Eyes Closed 3 | -3.498 | 0.009 |
| 34 | *12- YN7 – Visualization | 19- Post YN Eyes Closed 3 | -2.821 | 0.034 |
| 35 | *13- YN8 – Relaxation | 19- Post YN Eyes Closed 3 | -3.594 | 0.007 |
| 36 | *14- YN9 – Sankalpa | 1-Baseline Eyes Closed 1 | -2.713 | 0.041 |
| 37 | *14- YN9 – Sankalpa | 18- Post YN Eyes Closed 2 | -2.973 | 0.025 |
| 38 | *14- YN9 – Sankalpa | 19- Post YN Eyes Closed 3 | -4.051 | 0.002 |
| 39 | *14- YN9 – Sankalpa | 21- Post YN Eyes Closed Average | -2.742 | 0.039 |
| 40 | *15- YN10 – Awareness of breath | 19- Post YN Eyes Closed 3 | -3.432 | 0.01 |
| 41 | *16- YN11 – Ending of practice | 1-Baseline Eyes Closed 1 | -3.096 | 0.02 |
| 42 | *16- YN11 – Ending of practice | 18- Post YN Eyes Closed 2 | -3.355 | 0.012 |
| 43 | *16- YN11 – Ending of practice | 19- Post YN Eyes Closed 3 | -4.443 | 0.001 |
| 44 | *16- YN11 – Ending of practice | 21- Post YN Eyes Closed Average | -3.124 | 0.019 |

*From *Yoga nidra* © CD; ^#^Power Spectra Density (PSD)

**Table S3**- Significant post hoc results in Theta-1 Frequency Band during different EEG time bins from 1-21 i.e. during the entire *yoga nidra* (YN) recording session including pre *yoga nidra* or Baseline (EEG time bins 1-5), *yoga nidra* (EEG time bins 6-16) and post *yoga nidra* (EEG time bins 17- 21) in the subjects at various regions.

| Two EEG Time-bins (from 1 -21) in which significant result was found | | | Mean Difference (^#^PSD values in dB between EEG time bins) | P- value |
| --- | --- | --- | --- | --- |
| Central Theta-1 | | | | |
| 1 | *8- YN3 - Sankalpa | 17- Post YN Eyes Closed 1 | 1.966 | 0.034 |
| 2 | *8- YN3 - Sankalpa | 18- Post YN Eyes Closed 2 | 1.957 | 0.035 |
| 3 | *8- YN3 - Sankalpa | 19- Post YN Eyes Closed 3 | 1.982 | 0.033 |
| 4 | *8- YN3 - Sankalpa | 20- Post YN Eyes Closed 4 | 2.104 | 0.023 |
| 5 | *8- YN3 - Sankalpa | 21- Post YN Eyes Closed Average | 2.002 | 0.031 |
| 6 | *9- YN4 – Rotation of consciousness | *14- YN9 – Sankalpa | 2.001 | 0.041 |
| 7 | *9- YN4 – Rotation of consciousness | *16- YN11 – Ending of practice | 1.902 | 0.049 |
| 8 | *9- YN4 – Rotation of consciousness | 17- Post YN Eyes Closed 1 | 2.363 | 0.012 |
| 9 | *9- YN4 – Rotation of consciousness | 18- Post YN Eyes Closed 2 | 2.354 | 0.012 |
| 10 | *9- YN4 – Rotation of consciousness | 19- Post YN Eyes Closed 3 | 2.380 | 0.012 |
| 11 | *9- YN4 – Rotation of consciousness | 20- Post YN Eyes Closed 4 | 2.501 | 0.008 |
| 12 | *9- YN4 – Rotation of consciousness | 21- Post YN Eyes Closed Average | 2.399 | 0.011 |
| Parietal Theta-1 | | | | |
| 13 | *8- YN3 - Sankalpa | 18- Post YN Eyes Closed 2 | 1.890 | 0.042 |
| 14 | *8- YN3 - Sankalpa | 20- Post YN Eyes Closed 4 | 1.992 | 0.032 |
| 15 | *9- YN4 – Rotation of consciousness | 17- Post YN Eyes Closed 1 | 1.855 | 0.049 |
| 16 | *9- YN4 – Rotation of consciousness | 18- Post YN Eyes Closed 2 | 2.038 | 0.03 |
| 17 | *9- YN4 – Rotation of consciousness | 20- Post YN Eyes Closed 4 | 2.140 | 0.023 |
| 18 | *9- YN4 – Rotation of consciousness | 21- Post YN Eyes Closed Average | 1.956 | 0.038 |
| Temporal Theta-1 | | | | |
| 19 | *9- YN4 – Rotation of consciousness | 20- Post YN Eyes Closed 4 | 2.080 | 0.049 |
| Occipital Theta-1 | | | | |
| 20 | *9- YN4 – Rotation of consciousness | 18- Post YN Eyes Closed 2 | 2.683 | 0.014 |
| 21 | *9- YN4 – Rotation of consciousness | 19- Post YN Eyes Closed 3 | 2.259 | 0.038 |
| 22 | *9- YN4 – Rotation of consciousness | 20- Post YN Eyes Closed 4 | 2.625 | 0.016 |
| 23 | *9- YN4 – Rotation of consciousness | 21- Post YN Eyes Closed Average | 2.421 | 0.026 |
| Prefrontal Theta-1 | | | | |
| 24 | *16- YN11 – Ending of practice | 19- Post YN Eyes Closed 3 | -2.451 | 0.033 |

*From *Yoga nidra* © CD; ^#^Power Spectra Density (PSD)

**Table S4**- Significant post hoc results in Alpha-1 Frequency Band during different EEG time bins from 1-21 i.e. during the entire *yoga nidra* (YN) recording session including pre *yoga nidra* or Baseline (EEG time bins 1-5), *yoga nidra* (EEG time bins 6-16) and post *yoga nidra* (EEG time bins 17- 21) in the subjects at various regions.

| S. no | Two EEG Time-bins (from 1 -21) in which significant result was found | | Mean Difference (^#^PSD values in dB between EEG time bins) | P- value |
| --- | --- | --- | --- | --- |
| Central Alpha-1 | | | | |
| 1 | *8- YN3 - Sankalpa | 17- Post YN Eyes Closed 1 | -2.916 | 0.03 |
| 2 | *8- YN3 - Sankalpa | 18- Post YN Eyes Closed 2 | -2.850 | 0.034 |
| 3 | *9- YN4 – Rotation of consciousness | 17- Post YN Eyes Closed 1 | -3.486 | 0.011 |
| 4 | *9- YN4 – Rotation of consciousness | 18- Post YN Eyes Closed 2 | -3.420 | 0.012 |
| 5 | *9- YN4 – Rotation of consciousness | 19- Post YN Eyes Closed 3 | -3.070 | 0.025 |
| 6 | *9- YN4 – Rotation of consciousness | 21- Post YN Eyes Closed Average | -3.160 | 0.021 |
| Parietal Alpha-1 | | | | |
| 7 | 1-Baseline Eyes Closed 1 | *9- YN4 – Rotation of consciousness | 2.643 | 0.04 |
| 8 | *6- YN1 - Preparation | *9- YN4 – Rotation of consciousness | 2.650 | 0.043 |
| 9 | *8- YN3 - Sankalpa | 17- Post YN Eyes Closed 1 | -2.827 | 0.026 |
| 10 | *8- YN3 - Sankalpa | 18- Post YN Eyes Closed 2 | -2.607 | 0.04 |
| 11 | *9- YN4 – Rotation of consciousness | 17- Post YN Eyes Closed 1 | -3.147 | 0.015 |
| 12 | *9- YN4 – Rotation of consciousness | 18- Post YN Eyes Closed 2 | -2.927 | 0.023 |
| 13 | *9- YN4 – Rotation of consciousness | 19- Post YN Eyes Closed 3 | -2.625 | 0.042 |
| 14 | *9- YN4 – Rotation of consciousness | 21- Post YN Eyes Closed Average | -2.768 | 0.032 |
| Temporal Alpha-1 | | | | |
| 15 | *9- YN4 – Rotation of consciousness | 17- Post YN Eyes Closed 1 | -3.410 | 0.03 |
| 16 | *9- YN4 – Rotation of consciousness | 18- Post YN Eyes Closed 2 | -3.232 | 0.04 |
| 17 | *9- YN4 – Rotation of consciousness | 19- Post YN Eyes Closed 3 | -3.165 | 0.044 |
| 18 | *9- YN4 – Rotation of consciousness | 21- Post YN Eyes Closed Average | -3.110 | 0.048 |
| Occipital Alpha-1 | | | | |
| 19 | 1-Baseline Eyes Closed 1 | *9- YN4 – Rotation of consciousness | 2.987 | 0.048 |
| 20 | *8- YN3 - Sankalpa | 17- Post YN Eyes Closed 1 | -3.084 | 0.039 |
| 21 | *9- YN4 – Rotation of consciousness | 17- Post YN Eyes Closed 1 | -3.544 | 0.019 |
| 22 | *9- YN4 – Rotation of consciousness | 18- Post YN Eyes Closed 2 | -3157 | 0.037 |
| 23 | *9- YN4 – Rotation of consciousness | 21- Post YN Eyes Closed Average | -2.992 | 0.048 |

*From *Yoga nidra* © CD; ^#^Power Spectra Density (PSD)

**Table S5**- Significant post hoc results in Alpha-2 Frequency Band during different EEG time bins from 1-21 i.e. during the entire *yoga nidra* (YN) recording session including pre *yoga nidra* or Baseline (EEG time bins 1-5), *yoga nidra* (EEG time bins 6-16) and post *yoga nidra* (EEG time bins 17- 21) in the subjects at various regions.

| S. no | Two EEG Time-bins (from 1 -21) in which significant result was found | | Mean Difference (^#^PSD values in dB between EEG time bins) | P- value |
| --- | --- | --- | --- | --- |
| Central Alpha-2 | | | | |
| 1 | *8- YN3 - Sankalpa | *16- YN11 – Ending of practice | -2.210 | 0.042 |
| 2 | *8- YN3 - Sankalpa | 17- Post YN Eyes Closed 2 | -2.715 | 0.013 |
| 3 | *8- YN3 - Sankalpa | 18- Post YN Eyes Closed 2 | -2.618 | 0.016 |
| 4 | *8- YN3 - Sankalpa | 19- Post YN Eyes Closed 3 | -2.640 | 0.015 |
| 5 | *8- YN3 - Sankalpa | 20- Post YN Eyes Closed 4 | -2.528 | 0.02 |
| 6 | *8- YN3 - Sankalpa | 21- Post YN Eyes Closed Average | -2.625 | 0.016 |
| Occipital Alpha-2 | | | | |
| 7 | *8- YN3 - Sankalpa | 17- Post YN Eyes Closed 2 | -3.856 | 0.012 |
| 8 | *8- YN3 - Sankalpa | 18- Post YN Eyes Closed 2 | -4.010 | 0.009 |
| 9 | *8- YN3 - Sankalpa | 19- Post YN Eyes Closed 3 | -3.931 | 0.01 |
| 10 | *8- YN3 - Sankalpa | 20- Post YN Eyes Closed 4 | -3.558 | 0.02 |
| 11 | *8- YN3 - Sankalpa | 21- Post YN Eyes Closed Average | -3.839 | 0.012 |
| 12 | *9- YN4 – Rotation of consciousness | 17- Post YN Eyes Closed 2 | -3.336 | 0.032 |
| 13 | *9- YN4 – Rotation of consciousness | 18- Post YN Eyes Closed 2 | -3.489 | 0.025 |
| 14 | *9- YN4 – Rotation of consciousness | 19- Post YN Eyes Closed 3 | -3.411 | 0.028 |
| 15 | *9- YN4 – Rotation of consciousness | 21- Post YN Eyes Closed Average | -3.319 | 0.033 |
| 16 | *10- YN5 – Feeling of heaviness | 18- Post YN Eyes Closed 2 | -3.047 | 0.05 |

*From *Yoga nidra* © CD; ^#^Power Spectra Density (PSD)
