# Supplementary figures and images for: Electrophysiological Evidence of Local Sleep During Yoga Nidra Practice
Source: Front Neurol. 2022 Jul 12;13:910794. doi: 10.3389/fneur.2022.910794 (PMC9315270; doi:10.3389/fneur.2022.910794)

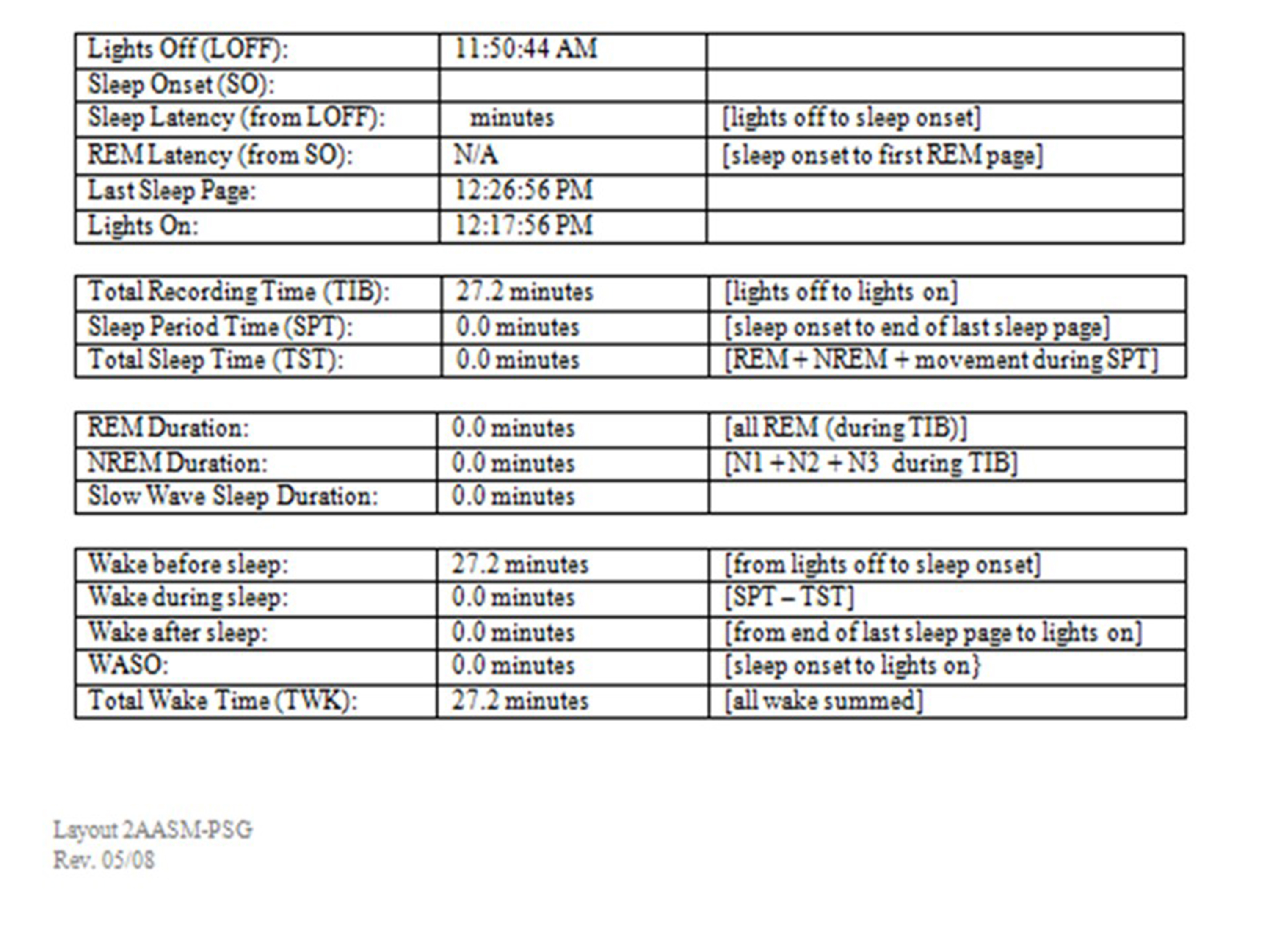

Supplement: Supplementary file 2 [file Image_1.JPEG]
